# Modeling of enzyme inactivation and quality changes during blanching of green beans

**DOI:** 10.1016/j.crfs.2026.101424

**Published:** 2026-05-16

**Authors:** Ruud G.M. van der Sman, Sophie M. Delbaere, Rian A.H. Timmermans

**Affiliations:** a Wageningen-Food & Biobased Research, The Netherlands; b Food Process Engineering, Wageningen University & Research, The Netherlands; c Ardo, Belgium

**Keywords:** Frozen vegetables, Thermal treatments, Enzyme, Texture, Modeling

## Abstract

Blanching is a critical step in the industrial production of frozen vegetables, primarily intended to inactivate enzymes and preserve quality during storage. Residual enzyme activity can cause undesirable changes in texture, flavor, and appearance. However, excessive blanching may also impair product quality. Therefore, understanding enzyme inactivation and its relation to quality deterioration under specific blanching conditions is essential.

This study developed models for the inactivation of selected enzymes during blanching of green beans prior to freezing. Additionally, if combined a heat-transfer the model can link the enzyme activity to texture development. Model validation was fitted using literature data and validated by new blanching experiments, measuring the activities of pectin methylesterase (PME), lipoxygenase (LOX), and peroxidase (POD).

Furthermore, it is shown that POD inactivation functions as a practical time–temperature integrator for blanching intensity, but LOX is even a more sensitive indicator of overall quality. Nevertheless, the equivalent-blanching-time correlated to POD activity, texture loss, and leaching metrics, suggesting it is a practical tool for blanching optimization.

## Highlights

• Development of predictive model for enzyme activity/inactivation during blanching.
• PME activity and beta-elimination determine texture development during blanching.
• POD enzyme is validated as time–temperature integrator, indicating blanching intensity.
• Effective time is a practical quantitative indicator for blanching intensity.

## Graphical abstract

**Figure:**
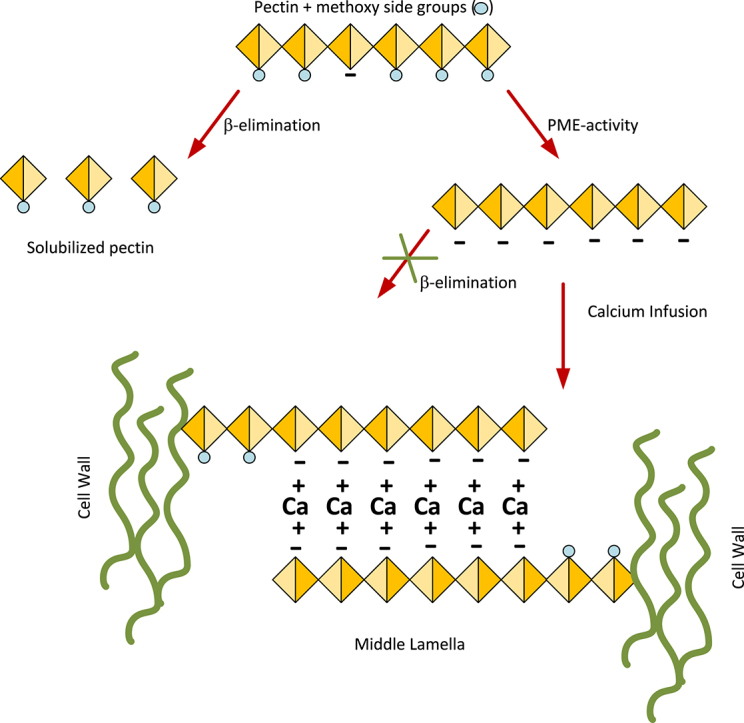

## 1 Introduction

The final eating quality of frozen vegetables – defined by texture, flavor, and nutritional value – is largely determined by industrial processing (Van der Sman, 2020). Among the most critical steps is blanching, which inactivates specific enzymes (Zhan et al., 2019). Without this inactivation, their biochemical reactions will continue during freezing, and they will even be amplified by the freeze-concentration effect (Van der Sman, 2020). Consequently, the quality of frozen foods would deteriorate rapidly during storage.

Besides its positive effects, excessive blanching can negatively impact product texture and nutritional content (Van Buggenhout et al., 2009). Prolonged blanching at temperatures above 90 °C promotes cell wall degradation through the β-elimination process, which solubilizes pectin (Verlinden and De Baerdemaeker, 1997, Van der Sman, 2020). This degradation leads to undesirable softening of texture. After blanching, vegetables should retain sufficient firmness and structure, as freezing already causes cellular damage and further texture loss (Jha et al., 2019). Next to texture changes, prolonged blanching also leads to loss of nutritional components via leaching into the blanching water.

Therefore, the blanching process for frozen vegetables must balance enzyme inactivation against texture degradation and loss of valuable nutrients (Richter Reis, 2016, van der Sman and Schenk, 2024). This optimization of blanching is currently difficult to achieve due to incomplete knowledge of all processes involved. This situation is not helped much with the little scientific interest in blanching nowadays. Most of the scientific studies orient on blanching with novel processing, (Jiang et al., 2020, Okonkwo et al., 2022, Astráin-Redín et al., 2023, Ahmed et al., 2026), with only limited optimization regarding inactivation of peroxidase (POD) or polyphenoloxidase (PPO) enzymes, often with the assumption of isothermal conditions. Only, a few studies on optimization of traditional hot water blanching (Pérez-Calderón et al., 2019, Pero et al., 2019) went a step further, combining enzyme inactivations models with heat transfer model. Yet, there is no study that includes texture degradation in the modeling.

**Fig. 1:**
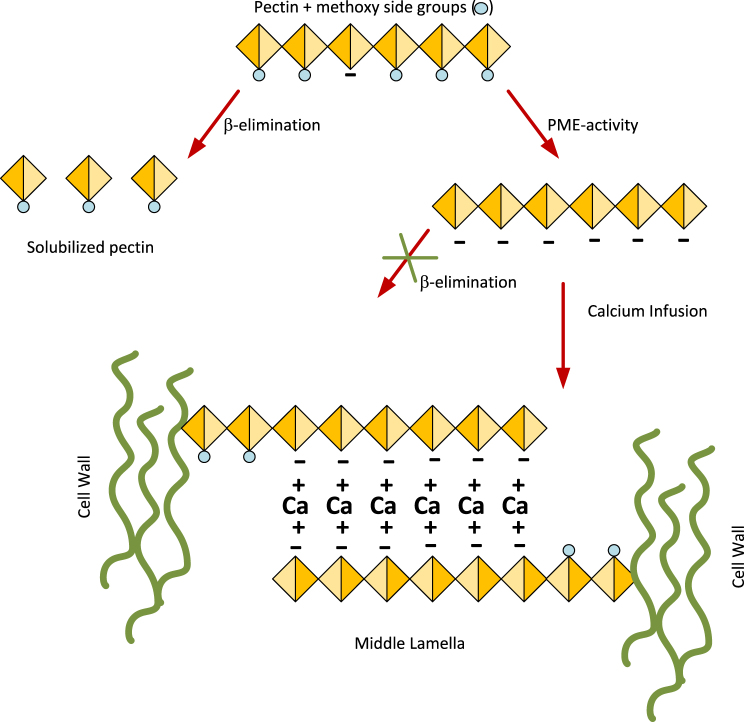
Schematic representation of chemical pathways for methoxylated pectin. β-elimination produces solubilized pectin. PME removes methyl groups, generating ionized carboxyl groups that are resistant to β-elimination. These carboxyl groups can bind divalent calcium ions, forming crosslinks in the middle lamella between adjacent cell walls.

Texture degradation during blanching is due to (a) loss of turgor, and (b) dissolution of pectin (Kunzek et al., 2002, Waldron et al., 2003, Van Buggenhout et al., 2009). Pectin is present in the middle lamella in between cells, and *glues* them together. Loss of pectin leads to a softening of the texture. While turgor loss is more or less inevitable, as cell membrane get ruptured already at temperatures above 40 °C, the dissolution of pectin is more amendable to control. Pectin dissolution is a thermal process, imparted by the so-called β-elimination process, which becomes significant at temperatures above 90 °C (Verlinden and De Baerdemaeker, 1997). However, if methyl-groups are removed from pectins, they become less sensitive to β-elimination. The removal of methyl groups is mediated by the PME (Pectin Methyl Esterase) enzyme. PME is an endogenous enzyme that removes ester groups from pectin — making it less susceptible to β-elimination (Fraeye et al., 2007). PME shows a maximal activity around 60 °C, but it is inactivated at temperatures exceeding 70 °C. Moreover, de-esterified pectin can crosslink with calcium, now increasing the vegetable texture. A schematic representation of these cell wall/pectin changes during blanching is shown in Fig. 1, which is adapted from Wolf and Greiner (2012).

There is only one single study which modeled both enzyme inactivation and texture changes during blanching (of carrots) by Verlinden and De Baerdemaeker (1997), but their model did not include the heat transfer from blanching water to the product. Nevertheless, their model for texture change is valuable, as it included both β-elimination, as well as the activity and inactivation of the enzyme PME. However, the interaction of pectin with calcium is absent in the model of Verlinden and De Baerdemaeker (1997). But, we view this existing framework as a good starting point for our study towards optimizing blanching of frozen vegetables, if it is extended with (1) relevant enzyme inactivation models, and (2) a heat transfer model.

In this study frozen green beans (*Phaseolus vulgaris L.*) is used as the case study for the optimization of the blanching process. For green beans, peroxidase (POD) and lipoxygenase (LOX) are considered key enzymes (Bahçeci et al., 2005). The heat-stable peroxidase (POD) is frequently studied, because it serves as an industrial indicator of blanching effectiveness (Halpin and Lee, 1987, Gökmen, 2010).

The role of POD as a key enzyme affecting the quality of many frozen vegetables has been debated (Williams et al., 1986). For green vegetables like green beans, LOX may play a more significant role as an quality indicator for the blanching process (Williams et al., 1986). Incomplete LOX inactivation can result in lipid oxidation, leading to the development of off-flavors during frozen storage. However, POD has been proposed as a time–temperature integrator to assess the impact of thermal processes such as blanching or sterilization on foods (Weng et al., 1991, Weng et al., 1992, Hendrickx et al., 1995). Hence, while not relevant for product quality, POD can still be a practical indicator for blanching intensity, as simple test are available for its inactivation — which are commonly used in food industry. Therefore, it is advised to develop inactivation models for both these enzymes. For the study of the optimization of blanching of green beans we develop a mathematical model describing (a) the inactvation of POD and LOX, (b) the activity and inactivation of PME, (c) β-elimination of pectin, as moderated by PME, and linked to texture, and (d) heat transfer. The heat transfer is computed using a Finite Volume model that describes the temporal and spatial changes of product temperature during blanching, which serves as input for the biochemical kinetics models.

The paper is organized as follows. First, models describing the biochemistry and heat transfer are introduced. Subsequently, the experimental setup is presented for tests performed to quantify heat transfer, enzyme inactivation, leaching and texture after blanching. The results section first presents the fitting of the biochemistry model to literature data and estimation of the heat transfer coefficient. Secondly, the fitted model is validated against own experimental data on enzyme inactivation, leaching and texture. Finally, the correlation between final quality traits and equivalent time – a practical measure of blanching intensity – is analyzed.

## 2 Model description

To describe cell wall modifications during blanching, the model originally developed for carrots by Verlinden and De Baerdemaeker (1997) is adopted. It is assumed that the underlying mechanisms are applicable to other vegetables, although parameter values may differ. The model includes the following reactions:

• Demethylation of pectin by PME,
• Denaturation of PME, and
• Depolymerization (solubilization) of pectin by β-elimination.

The three reactions are modeled as:

$$\partial_{t}\left[Pm\right]=-k_{\beta }\left[Pm\right]-k_{m}\left[Pm\right]\left[PME\right]$$

$$\partial_{t}\left[Pe\right]=+k_{m}\left[Pm\right]\left[PME\right]$$

(1) $$\partial_{t}\left[PME\right]=-k_{d}\left[PME\right]$$

[Pm] is the amount of methylated pectin, [Pe] is the amount of demethylated pectin, [PME] is the amount of enzyme. $k_{i}$ are the various reaction rates of the biochemical reactions.

In the original model all reaction rates are assumed to follow the Arrhenius relation:

(2) $$k_{i}=k_{i,ref}\exp\left(-\Delta E_{i}/R\left(\frac{1}{T}-\frac{1}{T_{ref}}\right)\right)$$

Furthermore, Verlinden and De Baerdemaeker (1997) assumed a linear relation assumed between non-solubilized pectin and firmness F. The force will be normalized against the initial maximal force. Hence, the normalized force equals:

(3) $$\overset{ˆ}{F}=\frac{\left[Pm\right]+\left[Pe\right]}{\left[Pm\left(0\right)\right]+\left[Pe\left(0\right)\right]}$$

[Pm(0)], and [Pe(0)] are the initial amounts of methylated/ demethylated pectin. It is assumed that initial degree of methylation equals:

(4) $$\left[DM\left(0\right)\right]=\frac{\left[Pm\left(0\right)\right]}{\left[Pm\left(0\right)\right]+\left[Pe\left(0\right)\right]}=0.8$$

following Canet et al. (2005).

The presence of methyl ester groups is assumed not to affect firmness in the absence of free calcium. Furthermore, turgor is assumed to be lost early in the blanching process, before any relevant enzyme is inactivated, and thus does not contribute to the firmness of blanched vegetables. Because no calcium is added to the blanching water, the effect of calcium is excluded from the model. Consequently, texture changes are attributed solely to pectin solubilization via the β-elimination process. However, this loss of texture can be modulated by PME activity, as demethylated pectin is considered resistant to β-elimination. Fraeye et al. (2007) reports that some chemical demethoxylation may occur at high temperatures under alkaline or acidic conditions, but this effect is not included in the model, since at high temperatures β-elimination dominates and PME activity is absent (Fraeye et al., 2007).

### 2.1 Enzyme inactivation kinetics

Models for the inactivation of POD, LOX, and PME are presented. The basic enzyme inactivation model assumes first-order kinetics with Arrhenius temperature dependence (Mcdonald and Bowman, 2018):

(5) $$\partial_{t}\left[Enz\right]=-k_{D,Enz}\left[Enz\right]$$

where the reaction rate follows $k_{D,Enz}∼\exp\left(-\Delta E_{Enz}/RT\right)$.

During blanching of many vegetables, multiple isozymes of POD and LOX are active, each exhibiting different inactivation kinetics (Güneş and Bayindirli, 1993). To account for this, a biphasic model is proposed (Połata et al., 2009):

(6) $$\partial_{t}\left[Enz_{I}\right]=-k_{D,EnzI}\left[Enz_{I}\right]\text{;}\partial_{t}\left[Enz_{II}\right]=-k_{D,EnzII}\left[Enz_{II}\right]$$

Here, $Enz_{I}$ and $Enz_{II}$ represent two isoforms of the enzyme acting on the same substrate but with distinct inactivation kinetics, governed by their respective rate constants $k_{D,EnzI}$ and $k_{D,EnzII}$.

### 2.2 Heat transfer model

Green beans typically have a diameter of about 9 mm and a length of approximately 5 cm. For modeling purposes, the geometry is approximated as an infinite cylinder. Heat transfer is described by the Fourier equation in cylindrical coordinates:

(7) $$\rho_{eff}c_{p,eff}\partial_{t}T=\frac{\lambda_{eff}}{r}\partial_{r}\left(r\partial_{r}T\right)$$

where $\rho_{eff}c_{p,eff}$ is the effective volumetric heat capacity and $\lambda_{eff}$ the thermal conductivity. These thermophysical properties depend on composition and are computed following the procedure in Van der Sman (2008). Compositional data from the USDA database are: 90% water, 7% carbohydrates, 2% proteins, and 0.6% ash. Thermal properties are assumed temperature-independent.

The Fourier equation is supplemented with the boundary condition:

(8) $$-\lambda_{eff}\partial_{r}T=h_{ext}\left(T-T_{0}\right)$$

where $h_{ext}$ is the heat transfer coefficient of the blanching medium and $T_{0}$ its temperature. The equation for a single green bean is solved using a cell-centered Finite Volume method with explicit Euler time integration. The bean is subdivided into five control volumes of equal size.

### 2.3 Goodness of model predictions

The goodness of the model predictions will be evaluated using the (normalized) Root Mean Square Error (RMSE):

(9) $$RMSE^{2}=\frac{1}{N}\overset{N}{\underset{i=1}{\sum }}{\left(y_{i}-{\overset{ˆ}{y}}_{i}\right)}^{2}$$

with $y_{i}$ the experimental data, and ${\overset{ˆ}{y}}_{i}$ the model prediction. N is the number of experimental datapoints. We will use interpolation (via Python interp1d function), to compute the model prediction at the time of the measurement. If the experimental dataset contains multiple temperatures, the RMSE will be averaged over all datapoints (of all temperatures).

### 2.4 Time–temperature integration

In industry, POD activity is widely used as an indicator of blanching intensity. Early studies recognized that enzymes can serve as convenient time–temperature integrators (Weng et al., 1991, Weng et al., 1992, Hendrickx et al., 1995), provided their inactivation follows first-order kinetics with Arrhenius temperature dependence. Process intensity can be quantified by an equivalent time at reference conditions (Hendrickx et al., 1995), defined as:

(10) $$t_{eff}=\int \exp\left(\frac{\Delta E_{a}}{R}\left(\frac{1}{T_{ref}}-\frac{1}{T}\right)\right)dt$$

Here, T denotes the product temperature, and $\Delta E_{a}$ is the activation energy for enzyme inactivation. The equivalent time represents the processing duration at a constant reference temperature that produces the same effect as the recorded dynamic temperature profile. In vegetables containing endogenous POD, the enzyme experiences the actual product temperature.

A similar measure for overall food quality decay was proposed by Dermesonluoglu et al. (2015):

(11) $$k_{ref}\left(T_{ref}\right)t_{eff}=\int_{0}^{t_{e}}k_{ref}\exp\left[\frac{\Delta E_{a}}{R}\left(\frac{1}{T_{ref}}-\frac{1}{T}\right)\right]dt$$

Here, $k_{ref}$ is the reaction rate coefficient at the reference temperature $T_{ref}$. The dimensionless form, $k_{ref}\left(T_{ref}\right)t_{eff}$, serves as an indicator of the intensity of non-isothermal treatments such as blanching or freezing. A similar concept was recently applied to describe firmness decay in avocados during postharvest logistics, where storage temperature and gas conditions vary (van der Sman et al., 2026).

In this study, the potential of an equivalent time as a practical alternative to the detailed biochemical model is evaluated. Specifically, it is examined whether quality decay can be predicted based on POD inactivation and whether POD can serve as a reliable indicator of blanching quality. Note that calculation of equivalent time requires the product temperature, which must be estimated using the heat transfer model if not directly measured.

**Fig. 2:**
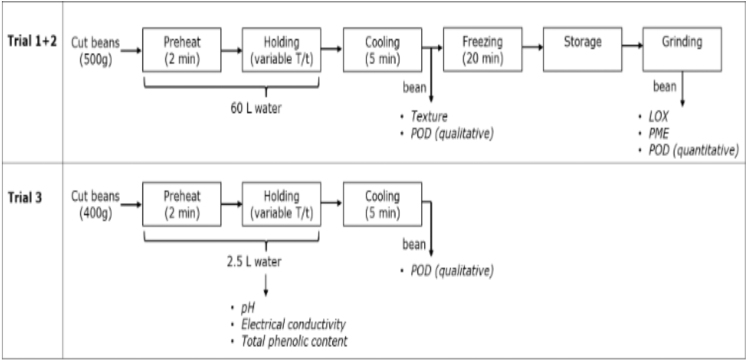
Schematic of the experimental set-up of our in-house experiments of blanching of green beans, combined with enzyme, texture and leaching analysis.

## 3 Materials and methods

### 3.1 Experimental set-up

Blanching experiments were conducted in three separate trials, using different green bean varieties and origins. In Fig. 2 a schematic representation of the blanching trials and performed analysis is shown.

Trial 1 was meant to establish enzyme inactivation kinetics across a broad temperature range. Haricot Vert Boby Tunnel beans (Morocco) were blanched in hot water at 35–96 °C for a fixed time. Each treatment consisted of 2 min heating to the target temperature, 5 min holding at the set temperature, followed by 5 min cooling in ice water. Peroxidase (POD) activity was measured immediately after blanching in qualitative method. Remaining samples were frozen, and analyzed later for lipoxygenase (LOX), pectin methylesterase (PME), and POD-activity (quantitative method).

Trial 2 has the goal to evaluate blanching conditions relevant to industrial practices and asses texture retention. Modesto beans (Netherlands), were blanched in hot water at 82–94 °C with 2 min preheating temperature, followed by holding times of 2–10 min. A portion of the beans was analyzed immediately for texture and qualitative POD activity. The remaining part was frozen for later determination of LOX, PME, and quantitative POD activities.

Trial 3 aims to evaluate the impact of blanching on leaching of bioactive compounds and water quality: Stanley beans (Netherlands) were blanched in hot water at 80–95 °C for 3–6.25 min holding time. POD activity was measured immediately after blanching. Blanching water was collected for analysis of leached compounds, including phenolics, pH and electrical conductivity.

### 3.2 Sample preparation and blanching

Fresh green beans were washed with cold water and cut into 5 cm segments. The mean weight of 150 individual segments was 3.32 g (range: 2.16–4.30 g), with an average diameter of 9.5 mm (range: 7.2–11.0 mm). For temperature monitoring, thermocouples (Amprobe, TMB-56 Multi-logging digital thermometer, equipped with thermocouples Type K) were inserted into the center and attached to the surface of two beans per batch. In Trials 1 & 2, 500 g of beans were heated in a water bath containing 60 L of water (type 1786S, nr 1946, Grosskuechen – Geraetebau Xanten/ANBO, Xanten, Germany). In Trial 3 400 g of beans were heated in a water bath of 2.5 L (Fisher Scientific, Polystat 37) to obtain more accurate leaching data. Temperature and time variations were applied according to the experimental design. After blanching, beans were immediately cooled in an ice-water bath for 5 min.

In Trials 1&2 a portion of the samples was analyzed directly for texture and POD activity, while the remainder was rapidly frozen in a shock freezer (type: P 0064-200010-10138-1, POOL Koudetechniek BV, Hengelo, The Netherlands) for 20 min to reach a temperature of -18 °C before storage. Frozen samples were stored at -18 °C. In Trial 3 beans were assessed only for POD activity after blanching. Blanching water was collected and analyzed for leached compounds.

Frozen green beans were homogenized under liquid nitrogen using a Grindomix at 10,000 rpm for 2 × 15 s (GM-type 200, Retsch, Haan, Germany) to obtain uniform samples for subsequent enzyme analysis.

### 3.3 Pectin methylesterase (PME) extraction and activity assay

For PME extraction, 50 g of homogenized frozen green beans were mixed with 150 mL demineralized water and stirred for 5 min at room temperature. The mixture was centrifuged for 20 min at 4700 rpm (Beckman Allegra X-30R Centrifuge), and the pellet was collected. To extract cell wall–bound PME, the pellet was resuspended in 0.2 M Tris–HCl buffer (pH 8.0) containing 1 M NaCl at a ratio of 1:1.3 (w/v). The suspension was mixed end-over-end for 60 min at room temperature, followed by centrifugation under the same conditions. The resulting supernatant was either used directly for PME activity analysis or stored at -18 °C until further use.

PME activity was determined based on the release of carboxyl groups from pectin. One unit of PME activity was defined as the amount of enzyme required to release 1μmol of COO${}^{-}$ groups per minute at 35 °C and pH 7.0, as described by Timmermans et al. (2022). For the assay, 1 mL of PME extract was added to 50 mL of 0.35% (w/v) apple pectin solution (degree of methylation: 70.3%) in 0.117 M NaCl at pH 6.5. The reaction mixture was maintained at 35 °C using a cryostat and stirred continuously. A pH-stat titration system (Metrohm, Herisau, Switzerland) automatically maintained the pH at 7.0 for 1200 s by adding 0.01 M NaOH. The rate of NaOH addition was used to calculate PME activity.

### 3.4 Lipoxygenase (LOX) extraction and activity assay

LOX was extracted from homogenized green bean powder following the methodology described by Rodrigo et al. (2007). For each extraction, 500 mg of frozen bean powder was suspended in 1.25 mL of extraction buffer (0.5 M Na${}_{2}$HPO${}_{4}$, pH 6.0) containing 0.5% (v/v) Tween 20 and 0.5% (v/v) Triton X-100. The mixture was placed in a 2 mL Eppendorf tube, kept on ice, and vortexed twice for 10 s. Samples were then incubated on a nutator at 4 °C while the substrate solution was prepared. After incubation, samples were centrifuged at 10,000 × g for 10 min at 4 °C. The supernatant was collected and used as the enzyme extract for LOX activity analysis.

LOX activity was measured based on the formation of conjugated dienes from linoleic acid, using sodium linoleate as the substrate. The substrate was prepared by mixing 84 mg of Tween 20 and 84 mg of linoleic acid with 4.8 mL of oxygen-free water, followed by addition of 0.72 mL of 1 M NaOH to clarify the solution. The final volume was adjusted to 30 mL with demineralized water.

The assay was performed at 25 °C in a quartz cuvette. The reaction mixture consisted of 2.7 mL of 0.2 M sodium phosphate buffer (pH 6.5), 30 μL of sodium linoleate substrate, and 100 μL of enzyme extract. Absorbance at 234 nm was recorded immediately and continuously for 10 min using a TECAN spectrophotometer. LOX activity was calculated from the linear portion of the absorbance curve. One unit of enzyme activity was defined as the amount of enzyme required to produce a change in absorbance of 1.0 per minute at 25 °C.

### 3.5 Guaiacol peroxidase (POD) extraction and activity assay

POD activity was determined by monitoring the oxidation of guaiacol to tetraguaiacol at 470 nm, following a modified method from Ruiz-Ojeda and Peñas (2013). The extinction coefficient for tetraguaiacol was 26.6 L mmol^−1^ cm^−1^.

For enzyme extraction, 0.4 g of ground frozen green bean tissue was mixed with 12 mL of 0.05 M phosphate buffer (pH 6.5) in a beaker and gently stirred for 5 min at room temperature. To initiate the reaction, 11.6 mL of assay solution – consisting of 0.001 M H${}_{2}$O${}_{2}$ and 0.003 M guaiacol prepared in 0.05 M phosphate buffer (pH 6.5) – was added to the extract. The reaction mixture was kept under constant stirring.

Aliquots were collected at several time points, filtered using 0.45 micrometer filters (surfactant-free cellulose acetate, 28 mm, Minisart, Sartorius, Göttingen, Germany) and transferred to cuvettes with a 10 mm optical path length (10 x 4 mm polystyrene, Sarstedt, Nümbrecht, Germany). Sampling times were recorded for each aliquot. Absorbance was measured at 470 nm using a Lambda 35 UV/Vis Spectrophotometer (PerkinElmer, Waltham, USA). POD activity was calculated from the slope of the absorbance versus time curve and expressed as the rate of tetraguaiacol formation, reflecting the remaining enzyme activity.

### 3.6 Qualitative peroxidase (POD) activity test

A rapid qualitative test was performed to assess residual POD activity in blanched green beans. This test is often used in food industry to assess blanching intensity. In this test ten beans per sample were cut longitudinally and arranged with the cut surface exposed. A test reagent consisting of 0.5% (w/v) guaiacol and 1.5% (w/v) hydrogen peroxide was sprayed uniformly onto the cut surfaces. The time until visible development of a red-brown coloration – indicating enzymatic oxidation of guaiacol – was recorded for each bean, up to a maximum of 180 s. Beans with high residual POD activity showed rapid color development within a few seconds, whereas longer discoloration times indicated lower remaining activity.

### 3.7 Texture analysis

A compression test was performed to determine the maximum force required to compress a single green bean. For each blanching condition, 15 individual beans were tested, and the average force was calculated. For the measurements a texture analyzer is used, TA.XTPlus, Stable Micro Systems, equipped with a 30 kg load cell, calibrated at 5 kg. A 75 mm compression plate (SMS P/75, Stable Micro Systems) was used as a probe for the measurements. The probe started from a height of 13 mm above the test platform, with a compression speed of 1.0 mm/s and a trigger force of 0.5 N to initiate contact with the sample. The sample was compressed 25% down, and the maximum force recorded during compression was used as an indicator of bean firmness.

### 3.8 Leaching indicators

Blanching water collected from Trial 3 was analyzed to assess the extent of compound leaching during the thermal process. Measurements were done with a pH meter with glass electrode (type 913, Metrohm) and an electrical conductivity meter (type FiveEasy FE30, Mettler Toledo). Total phenolic content (TPC) of blanching water samples was determined using the Folin–Ciocalteu method, based on the reduction of the Folin–Ciocalteu reagent by phenolic compounds under alkaline conditions, resulting in the formation of a blue chromophore measurable spectrophotometrically, following a modified procedure described by Adedeji et al. (2025).

The Folin–Ciocalteu reagent (Sigma-Aldrich) was diluted tenfold (1:10, v/v) with distilled water prior to use. An aliquot of 100 μL of the diluted sample was transferred into test tubes (in triplicate). For the blank, 100 μL distilled water was used. Subsequently, 750 μL of diluted Folin–Ciocalteu reagent was added to each tube, and the mixtures were allowed to react for 5 min. Thereafter, 750 μL of Na${}_{2}$CO${}_{3}$ solution was added to establish alkaline conditions. The mixtures were vortexed and incubated in the dark at room temperature for 60 min to allow full color development. Absorbance was measured at 765 nm using a UV–Vis spectrophotometer (Hach Lange DR 3900, Germany) equipped with quartz cuvettes (optical path length: 10 mm). A calibration curve was constructed using Gallic acid (Thermo Fisher Scientific) as the reference standard, and results were expressed as mg gallic acid equivalents per liter of blanching water (mg GAE/L).

**Fig. 3:**
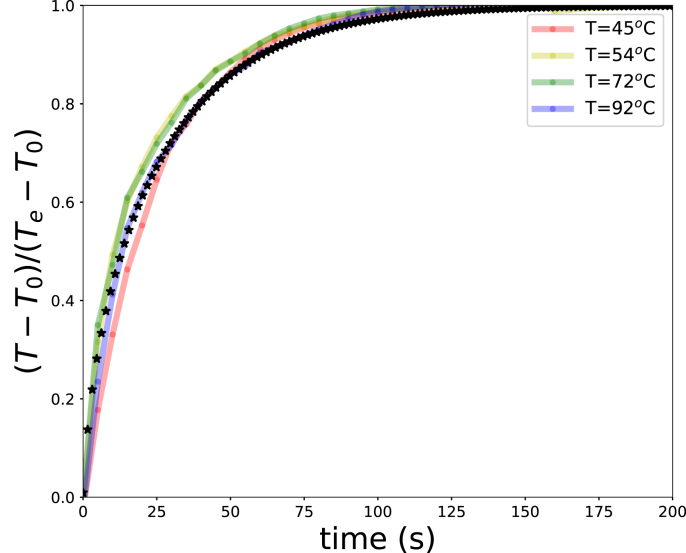
Average green bean temperature during hot-water blanching with h=1000 W m^−2^ K^−1^ at different bath temperatures. Experimental data are shown as reduced temperatures; simulation results are indicated by star symbols.

## 4 Results and discussion

### 4.1 Heat transfer model

The heat transfer model was validated against temperature measurements of green bean pods during blanching in hot-water baths. Data from experiment 1 were used, performed at bath temperatures of $T_{e}=42$, 54, 72, and 92 °C. First, the temperature data were normalized using the following rescaling:

(12) $$\theta =\frac{T-T_{0}}{T_{e}-T_{0}}$$

where $T_{0}\approx 25\;{}^{\circ}C$ is the initial temperature and $T_{e}$ the bath temperature. After rescaling, the experimental data collapsed to a single curve (Fig. 3), consistent with the assumption of temperature-independent thermal properties. From the heat transfer model, the average temperature was extracted and fitted to the experimental data by varying the heat transfer coefficient. Good agreement was obtained for $h_{ext}=1000$ W m^−2^ K^−1^, as shown in Fig. 3, which aligns reasonably with values reported in literature (Lamberg and Hallström, 1986, Iribe-Salazar et al., 2015).

Fig. 3 shows that within 2 min the average temperature approaches the blanching temperature. Given that typical residence times of green beans in industrial blanchers are about 500 s, internal heat conduction within the bean is expected to significantly influence enzyme inactivation.

### 4.2 Fitting biochemistry models against literature data

#### 4.2.1 LOX inactivation

The biphasic first-order model was used to describe LOX inactivation in green beans, following Indrawati et al. (2000). The model was fitted to isothermal data reported by Indrawati et al. (2000). In these experiments, the enzyme was first extracted at room temperature and subsequently subjected to heat treatments, with activity analyzed after heating. It is assumed that the enzyme experienced isothermal conditions during these tests. Fitting the model to this dataset is advantageous, because enzymes always follows the bath temperatures, while for whole beans the enzyme model needs to be coupled to heat transfer model - introducing more uncertainty.

**Fig. 4:**
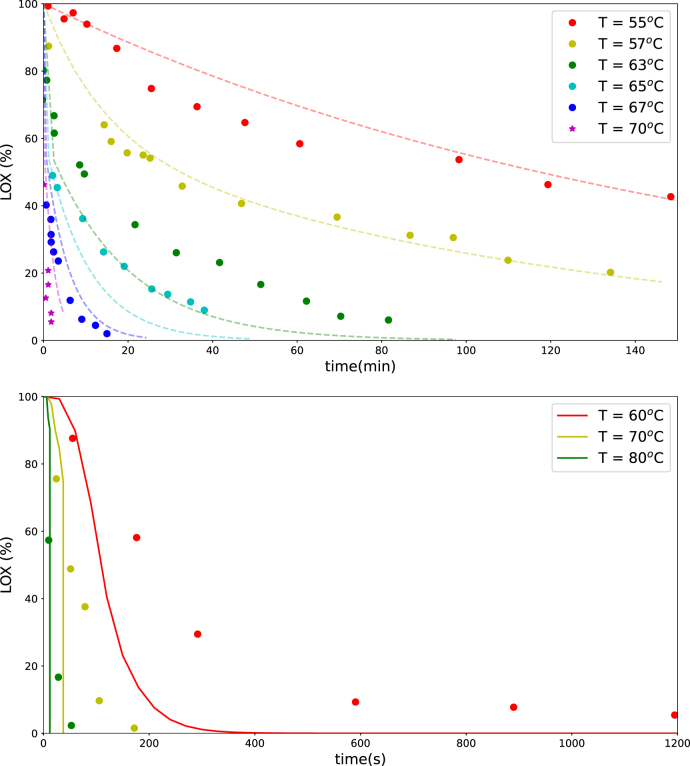
Predicted remaining LOX activity in blanched green beans based on the biphasic first-order model (lines), compared with isothermal experimental data from Indrawati et al. (2000) (top) and non-isothermal data from Bahçeci et al. (2005) (bottom) for various blanching temperatures and times.

Parameter estimation for the biphasic model was performed using the SciPy optimization routine with the *L-BFGS-B* method and a tolerance of 10^−4^. The $L_{2}$ norm for different experiments was weighted by the number of data points in each experiment. Results are shown in the top panel of Fig. 4. Optimal fitting was obtained with the following parameter values: $Enz_{I}\left(0\right)/Enz_{II}\left(0\right)=A_{I}=0.53$, $\Delta E_{I}=282$ kJ/mol, $\Delta E_{II}=96$ kJ/mol, $k_{D,I,ref}=0.36\times 10^{-3}$ s^−1^, and $k_{D,II,ref}=0.028$ s^−1^. Model predictions agree well with experimental data for T=55, 57, 67, and 70 °C, whereas predictions for 63 and 65 °C deviate, likely due to inconsistencies in the experimental data.

Validation of the fitted model was performed against the data of Bahçeci et al. (2005). These data were obtained for whole beans, meaning the enzymes experienced non-isothermal conditions. The temperature profile within the beans was computed using the above fitted heat transfer model. For each control volume, enzyme inactivation was calculated, and the average value was compared to the experimental data of Bahceci.

Results are shown in Fig. 4. Model predictions provide a reasonable match to the Bahceci dataset, with a RMSE of 0.22, supporting the validity of the LOX inactivation model.

The discrepancy between model prediction and Bahceci data is a symptom of the general problem of variability amongst different literature sources. To illustrate that point in the Supplementary Material we have listed activation energies $\Delta E_{a}$ of POD/LOX/PME enzymes in various vegetables. For a single vegetable one finds wide variations in values, but sometimes comparable to other vegetables. Some universality in $\Delta E_{a}$ amongst vegetables can be expected, but variations can also be induced by differences in experimental methods and enzyme assays used. Consequently, the ultimate test of the model fit is the comparison against our own experimental data, as discussed below.

#### 4.2.2 POD inactivation

POD inactivation was modeled using experimental data on whole green beans from Bahçeci et al. (2005), the same source used for validating the LOX model. As in the LOX case, a biphasic first-order model coupled to the Fourier equation was applied. Because the biphasic model contains five parameters, automated fitting showed poor convergence, and the *L-BFGS-B* method became trapped in a local minimum. Consequently, parameter estimation was performed manually. In the manual fitting we aimed to minimize the $L_{2}$ norm between experiments and predictions. We varied one parameter at a time, choose the value that minimized $L_{2}$, and proceed to the next parameter. This cycle was performed several times, until the change in $L_{2}$ was less then 0.5%.

**Fig. 5:**
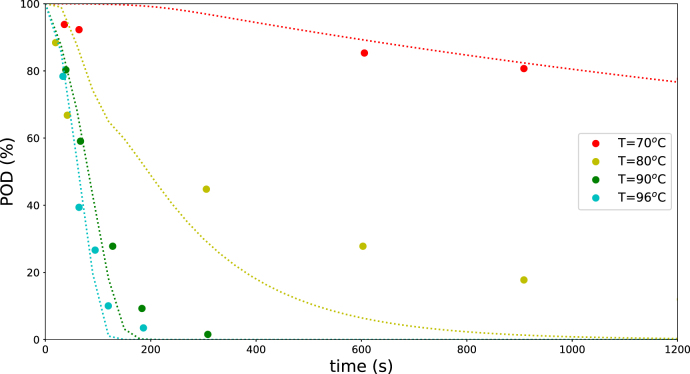
Remaining POD activity in blanched green beans predicted by the biphasic first-order model (dotted lines), compared with experimental data from Bahçeci et al. (2005) for various blanching temperatures and times.

The fitted parameter values are $A_{I}=Enz_{I}\left(0\right)/\left(Enz_{I}\left(0\right)+Enz_{II}\left(0\right)\right)=0.70$, $k_{I,ref}=12\times 10^{-5}$ s^−1^, $k_{II,ref}=6.8\times 10^{-4}$ s^−1^, $\Delta E_{I}=0.37$ MJ/mol, and $\Delta E_{II}=4.7$ MJ/mol. The results, shown in Fig. 5, indicate that the biphasic model captures the overall trends in the dataset reasonably well despite the challenges in parameter estimation.

#### 4.2.3 PME inactivation

**Fig. 6:**
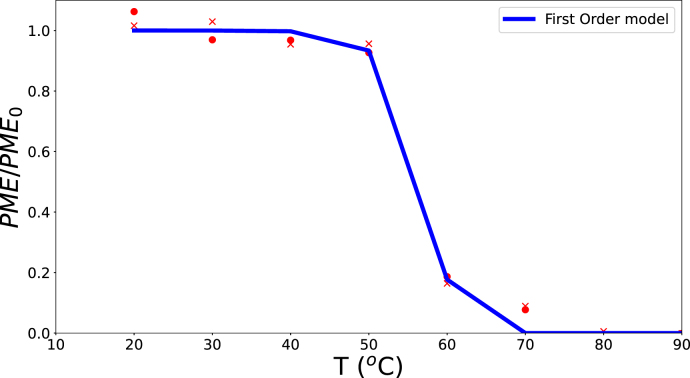
Remaining PME activity in blanched green beans at various temperatures and blanching times, using experimental data from Stolle-Smits et al. (2000). Predictions from the monophasic first-order model are shown as solid lines.

PME inactivation was modeled using experimental data on whole green beans from Stolle-Smits et al. (2000). The data were fitted with a monophasic first-order model coupled to the Fourier equation, using the *L-BFGS-B* optimization method. A good agreement was obtained with parameter values $k_{D,PME,ref}=91\times 10^{-3}$ s^−1^ and $\Delta E_{PME}=315$ kJ/mol, as shown in Fig. 6.

### 4.3 PME activity

**Fig. 7:**
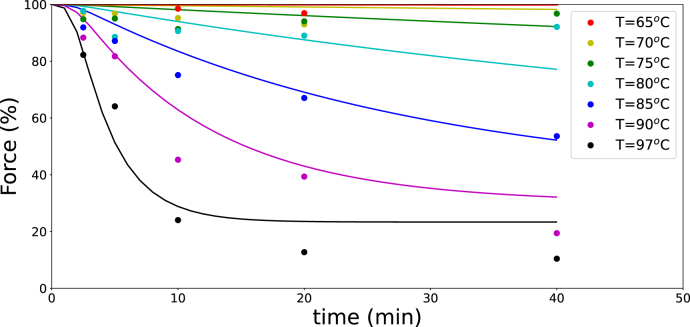
Texture measurements of blanched whole green beans at various blanching temperatures, using experimental data from Canet et al. (2005), compared with predictions from the Verlinden and De Baerdemaeker (1997) model.

As a validation of the Verlinden and De Baerdemaeker (1997) model, predictions of texture were compared with experimental data on whole green beans from Canet et al. (2005). A linear relation was assumed between texture loss and the amount of solubilized pectin. The results are presented in Fig. 7.

By combining the Verlinden and De Baerdemaeker (1997) model with the heat-transfer model, the predicted texture values were fitted to the normalized force measurements. The estimated parameter values for β-elimination and PME activity are: $k_{\beta }\left(T_{ref}=373,\text{K}\right)=0.2\times 10^{-3}$ s^−1^, $\Delta E_{\beta }=200$ kJ/mol, $k_{PME}\left(T_{ref}=338,\text{K}\right)=0.011$ s^−1^, and $\Delta E_{PME}=100$ kJ/mol. With these values, the model provides a reasonable description of the experimentally measured texture.

### 4.4 Validation of inactivation models against in-house data

The validity of the enzyme inactivation models was assessed using in-house experimental data. Green beans were blanched for 5 min over a range of temperatures, and the residual activities of POD, LOX, and PME were measured. These measurements were compared with model predictions based on parameter values estimated from literature data, as described in the previous subsections.

**Fig. 8:**
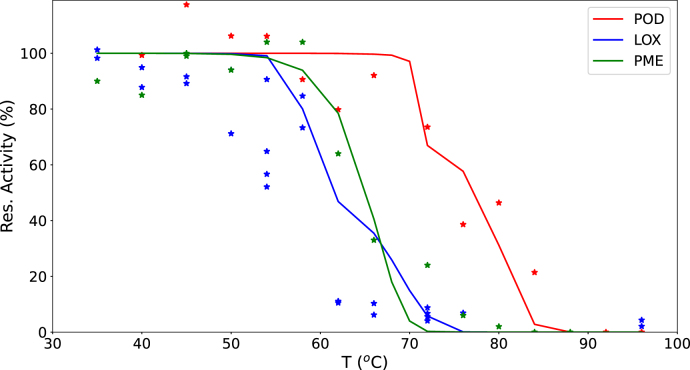
Predicted remaining enzyme activity in blanched green beans (lines) compared with in-house experimental data (symbols with matching colors), obtained by blanching for 5 min at various temperatures.

Experimental data were normalized by setting the activity at 35 °C to 100%. Fig. 8 presents the comparison between model predictions and experimental results. The experiments were performed on whole beans, and the product temperature evolution was computed using the Fourier equation. The results in Fig. 8 show that the model predictions agree reasonably well with the experimental data, as shown by the RMSE of 17.4%, 20.7%, and 9.9% for POD, LOX and PME respectively. This indicates that the estimated parameter values are applicable across different batches of green beans, even when originating from different harvest years.

**Fig. 9:**
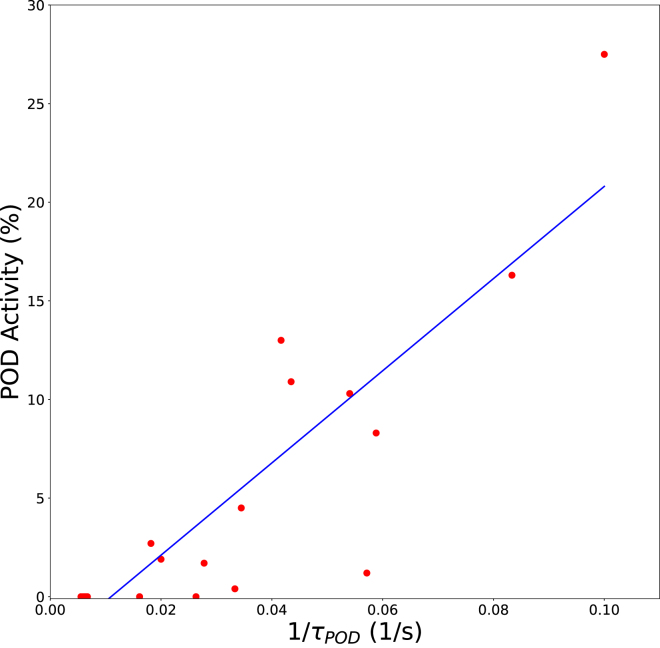
Correlation between residual POD activity (quantitative assay) and the inverse reaction time of the qualitative POD peroxide test ($1/\tau_{POD}$). The blue line represents a linear regression fit ($r^{2}=0.77$).

Additional blanching experiments (denoted as Experiment 2 in M&M) were conducted to reflect industrial practice, incorporating a range of blanching temperatures and residence times. POD activity was assessed using two methods: a quantitative POD assay and the qualitative POD peroxide test commonly used in industry. The qualitative test results were compared with POD inactivation measurements obtained from the assay. The peroxide test measures the reaction time τ required for color development, which was assumed to be inversely proportional to the reaction rate and, consequently, to the residual POD activity. This assumption was evaluated by comparing the residual POD activity from the assay with 1/τ, as shown in Fig. 9. Statistical analysis yields a correlation coefficient of $r^{2}=0.77$, supporting the usefulness of the qualitative POD test as an indicator of POD inactivation.

**Fig. 10:**
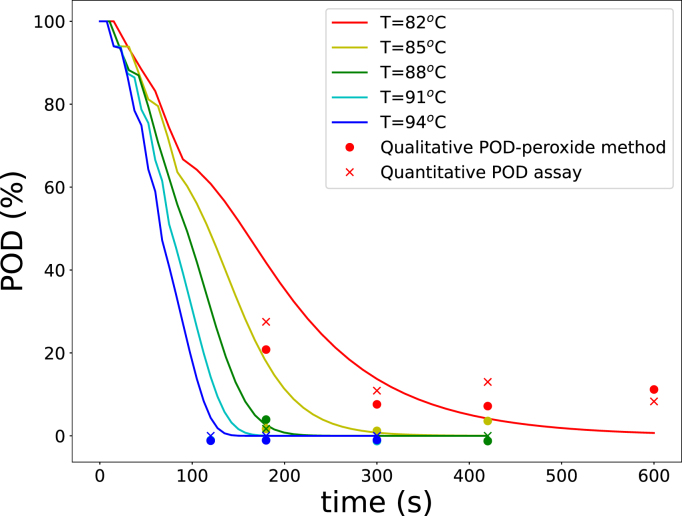
Comparison of predicted POD activity with experimental data obtained from lab-scale experiments, using both the qualitative and quantitative POD methods.

Lab-scale experiments showed substantial residual POD activity after blanching, whereas LOX and PME activities were negligible. Model predictions of POD inactivation were compared with experimental measurements from both analytical methods (Fig. 10). Qualitative POD peroxide test results were converted to residual POD activity using the correlation established in Fig. 9. The model exhibited good agreement with the experimental data for both measurement approaches, as expressed in the RMSE of 8.5% and 7.1% for the qualitative and quantitative POD methods, confirming the models applicability for describing POD inactivation under non-isothermal conditions.

**Fig. 11:**
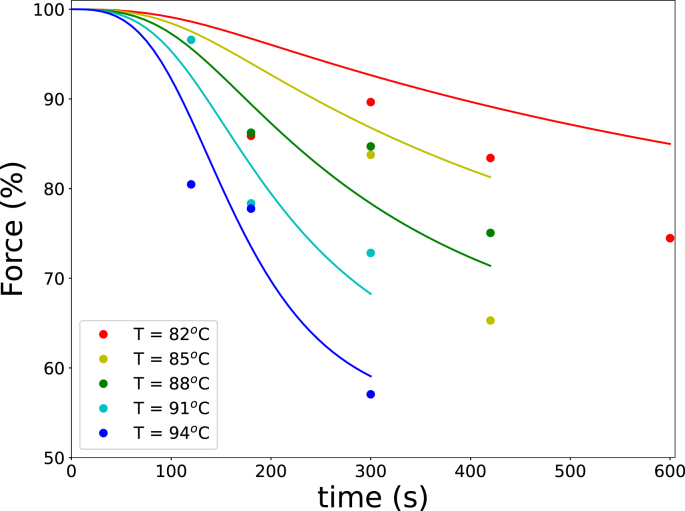
Comparison of predicted texture (lines) with experimental data (symbols) obtained from the lab-scale experiments.

Finally, texture predictions were validated against force measurements (Fig. 11), calculated using Eq. (3). The texture predictions capture the overall trends in texture changes, as evidenced by the RMSE=7.8%, demonstrating its suitability for describing blanching-induced quality modifications.

### 4.5 Evaluation of the equivalent time concept

The applicability of the equivalent time concept was evaluated by examining whether POD inactivation (Fig. 10) and texture changes after blanching (Fig. 11) could be represented on a single curve when plotted against the equivalent time $t_{eff}$. The activation energy $\Delta E_{a}$ was optimized separately for each case.

**Fig. 12:**
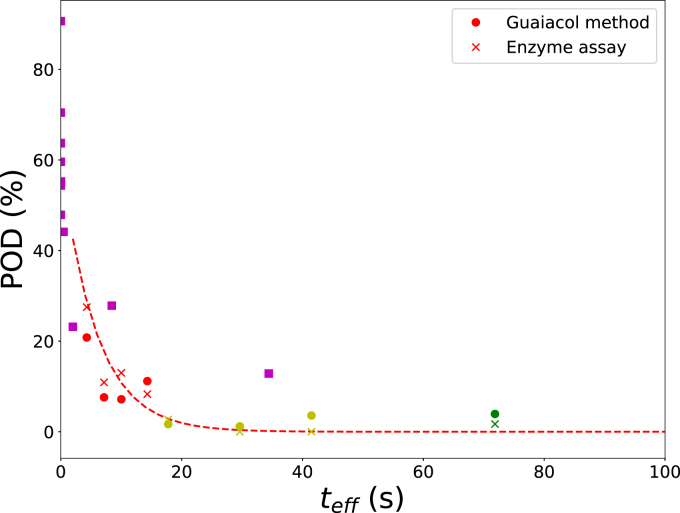
Residual POD activity from the experiments, including data from Fig. 8, plotted against the equivalent time $t_{eff}$, calculated using Eq. (9) with $\Delta E_{a}=\Delta E_{I}=0.37$ MJ/mol. The dashed line shows the prediction of a monophasic first-order inactivation model with $\Delta E_{a}=\Delta E_{I}$ and $A_{I}=0.60$. The initial steep decline reflects the rapid inactivation of the heat-labile fraction.

**Fig. 13:**
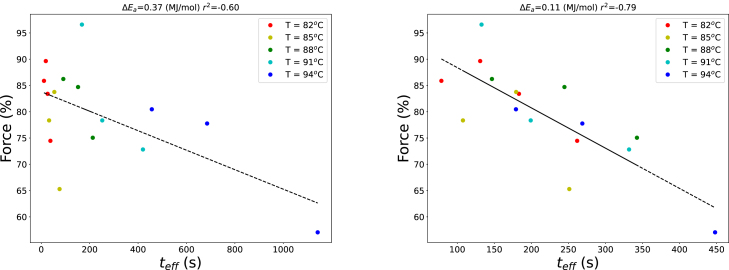
Measured texture of green beans, expressed as relative compression force, plotted against the equivalent time using $\Delta E_{a}=\Delta E_{I}$ (left panel) and $\Delta E_{a}=0.11$ MJ/mol, which yields the maximum $r^{2}$ (right panel).

Initially, the activation energy corresponding to the heat-stable fraction of POD ($\Delta E_{a}=\Delta E_{I}=0.37$ MJ/mol) was used to compute $t_{eff}$. Residual POD activity was then plotted against equivalent time, along with the theoretical decay predicted by a monophasic first-order kinetic model using the same activation energy (Fig. 12). Results from both the quantitative enzyme assay and the guaiacol-based qualitative test closely followed the theoretical curve, indicating that POD inactivation can be effectively represented using the equivalent time approach that integrates the time–temperature history. Texture measurements, expressed as relative compression force, were also plotted against equivalent time, as shown in figure Fig. 13.

Using $\Delta E_{a}=\Delta E_{I}$ resulted in a moderate correlation ($r^{2}=0.60$). Subsequent optimization showed that $\Delta E_{a}=0.11$ MJ/mol improved the correlation to $r^{2}=0.80$. This activation energy is approximately equal to reported values for pectin hydrolysis catalyzed by PME or for LOX inactivation. These findings indicate that POD provides a reasonable measure of the time–temperature history during blanching, whereas LOX may serve as a more suitable indicator of product quality (Williams et al., 1986, Barrett and Theerakulkait, 1995). Although no practical LOX test comparable to the guaiacol assay exists in industrial practice, several simple colorimetric LOX assays for crude vegetable extracts have been reported in the literature (Anthon and Barrett, 2001, Reyes-De-Corcuera et al., 2002, De Corcuera et al., 2003).

Henceforth, the industrial usage of POD enzyme with the (qualitative) guaiacol test as blanching-intensity indicator should be considered with care, because of the moderate correlation of the equivalent time with product quality if one assumes $\Delta E_{a}=\Delta E_{I}$, i.e. the activation energy for inactivating POD. For green beans it is adviced to develop an industrial test based on LOX. Alternatively, the guaiacol POD test should be transformed into a more quantitative test with more precise assessment of the reaction time, $\tau_{POD}$, and use a non-linear mapping of the reaction time to food quality. For this mapping models as presented in this paper can be used.

### 4.6 Results from leaching experiments

**Fig. 14:**
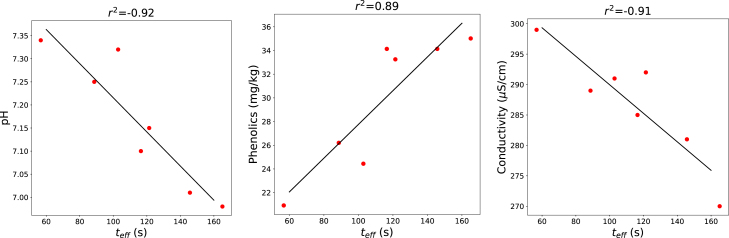
Leaching measurements (symbols) plotted against the equivalent time $t_{eff}$ using $\Delta E_{a}=0.11$ MJ/mol. Solid lines represent linear regression fits. The corresponding correlation coefficients ($r^{2}$) are shown in each panel.

During the leaching experiments, green beans were immersed in hot water baths for defined times and temperatures. The pH, conductivity, and phenolic content of the blanching water were measured as indicators of compounds leached from the beans. Using the heat-transfer model, the evolution of product temperature was calculated, and the equivalent time was estimated using $T_{ref}=90\;{}^{\circ}C$ and $\Delta E_{a}=0.11$ MJ/mol. Note, we have assumed that the leaching of solids did not influence the heat transfer rate. Data from the leaching tests are presented in Fig. 14. Linear regression of the leaching metrics against the equivalent time shows a strong correlation ($r^{2}\approx 0.9$), indicating that equivalent time is a suitable predictor of leaching during blanching.

**Table A.1:** LOX/PME inactivation parameters of various vegetables amongst literature.

Asparagus	LOX biphasic	$\Delta E_{a,I}$	76 kJ/mol	Morales-Blancas et al. (2002)
Asparagus	LOX biphasic	$\Delta E_{a,II}$	65 kJ/mol	Morales-Blancas et al. (2002)
Broccoli	LOX biphasic	$\Delta E_{a,I}$	108.3 kJ/mol	Pérez-Calderón et al. (2017)
Broccoli	LOX biphasic	$\Delta E_{a,II}$	34.6 kJ/mol	Pérez-Calderón et al. (2017)
Broccoli	LOX biphasic	$\Delta E_{a,I}$	61 kJ/mol	Morales-Blancas et al. (2002)
Broccoli	LOX biphasic	$\Delta E_{a,II}$	55 kJ/mol	Morales-Blancas et al. (2002)
Brussel sprouts	LOX biphasic	$\Delta E_{a,I}$	68.5 kJ/mol	Pérez-Calderón et al. (2017)
Brussel sprouts	LOX biphasic	$\Delta E_{a,II}$	63.7 kJ/mol	Pérez-Calderón et al. (2017)
Green beans	LOX monophasic	$\Delta E_{a}$	160 kJ/mol	Garrote et al. (2001)
Green Beans	LOX biphasic	$\Delta E_{a,I}$	198 kJ/mol	Güneş and Bayindirli (1993)
Green Beans	LOX biphasic	$\Delta E_{a,II}$	57 kJ/mol	Güneş and Bayindirli (1993)
Peas	LOX biphasic	$\Delta E_{a,I}$	207 kJ/mol	Güneş and Bayindirli (1993)
Peas	LOX biphasic	$\Delta E_{a,II}$	46 kJ/mol	Güneş and Bayindirli (1993)
Carrot	PME biphasic	$\Delta E_{a,I}$	635 kJ/mol	Anthon and Barrett (2002)
Carrot	PME biphasic	$\Delta E_{a,II}$	510 kJ/mol	Anthon and Barrett (2002)
Cauliflower	PME monophasic	$\Delta E_{a}$	208 kJ/mol	Rayan et al. (2011)
Potato	PME biphasic	$\Delta E_{a,I}$	759 kJ/mol	Anthon and Barrett (2002)
Potato	PME biphasic	$\Delta E_{a,II}$	493 kJ/mol	Anthon and Barrett (2002)

## 5 Conclusions

This study developed and validated mechanistic models describing enzyme inactivation and texture degradation in green beans during blanching. Biphasic first-order kinetics were used for POD and LOX, while PME inactivation was captured with a monophasic model. These biochemical models were coupled to a heat-transfer model based on the Fourier equation to account for non-isothermal conditions. Validation against literature data and lab-scale experiments showed good agreement between model predictions and measured enzyme activities and texture changes, demonstrating the robustness of the estimated parameters across different batches and harvest years.

Further analysis indicated that POD inactivation can function as a practical time–temperature integrator for blanching intensity, whereas LOX may be a more sensitive indicator of overall quality. The equivalent time concept was successfully applied to POD activity, texture loss, and leaching metrics, suggesting its usefulness as a simplified tool for process optimization. These results provide a basis for designing blanching processes that balance microbial safety, enzyme inactivation, and texture preservation, thereby enhancing product quality and energy efficiency in industrial applications.

## Declaration of competing interest

One of the authors is employed by company who partly sponsored the project financially. We declare that had no influence on the scientific results of this study.

## Figures and Tables

**Table A.2:** POD inactivation parameters of various vegetables amongst literature.

Asparagus	POD biphasic	$\Delta E_{a,I}$	67 kJ/mol	Morales-Blancas et al. (2002)
Asparagus	POD biphasic	$\Delta E_{a,II}$	43 kJ/mol	Morales-Blancas et al. (2002)
Broccoli	POD biphasic	$\Delta E_{a,I}$	75 kJ/mol	Morales-Blancas et al. (2002)
Broccoli	POD biphasic	$\Delta E_{a,II}$	58 kJ/mol	Morales-Blancas et al. (2002)
Broccoli	POD biphasic	$\Delta E_{a,I}$	71.2 kJ/mol	Pérez-Calderón et al. (2017)
Broccoli	POD biphasic	$\Delta E_{a,II}$	66.9 kJ/mol	Pérez-Calderón et al. (2017)
Broccoli	POD monophasic	$\Delta E_{a}$	159 kJ/mol	Gonçalves et al. (2009)
Brussel sprouts	POD biphasic	$\Delta E_{a,I}$	62.5 kJ/mol	Pérez-Calderón et al. (2017)
Brussel sprouts	POD biphasic	$\Delta E_{a,II}$	56.3 kJ/mol	Pérez-Calderón et al. (2017)
Cauliflower	POD monophasic	$\Delta E_{a}$	156 kJ/mol	Rayan et al. (2011)
Carrot	POD biphasic	$\Delta E_{a,I}$	95 kJ/mol	Morales-Blancas et al. (2002)
Carrot	POD biphasic	$\Delta E_{a,II}$	86 kJ/mol	Morales-Blancas et al. (2002)
Carrot	POD biphasic	$\Delta E_{a,I}$	57 kJ/mol	Güneş and Bayindirli (1993)
Carrot	POD biphasic	$\Delta E_{a,II}$	52 kJ/mol	Güneş and Bayindirli (1993)
Carrot	POD monophasic	$\Delta E_{a}$	480 kJ/mol	Anthon and Barrett (2002)
Carrot	POD Biphasic	$\Delta E_{a,I}$	148 kJ/mol	Soysal and Söylemez (2005)
Carrot	POD Biphasic	$\Delta E_{a,II}$	90 kJ/mol	Soysal and Söylemez (2005)
Green Beans	POD biphasic	$\Delta E_{a,I}$	77 kJ/mol	Güneş and Bayindirli (1993)
Green Beans	POD biphasic	$\Delta E_{a,II}$	57 kJ/mol	Güneş and Bayindirli (1993)
Green Beans	POD monophasic	$\Delta E_{a}$	48 kJ/mol	Ruiz-Ojeda and Peñas (2013)
Peas	POD biphasic	$\Delta E_{a,I}$	75 kJ/mol	Güneş and Bayindirli (1993)
Peas	POD biphasic	$\Delta E_{a,II}$	41 kJ/mol	Güneş and Bayindirli (1993)
Potato	POD monophasic	$\Delta E_{a}$	478 kJ/mol	Anthon and Barrett (2002)

## Appendix Activation energy of enzyme inactivation

See Table A.1, Table A.2.

## References

[b1] Adedeji F.T., Adeyanju A.A., Bamidele O.P. (2025). Phenolic profile, physicochemical properties and antioxidant activities of blanching water obtained from some green leafy vegetables. Food Chem. Adv..

[b2] Ahmed J.M., Mowafy S., Guo J., Okaiyeto S.A., Yu S.-H., Liu Y. (2026). High humidity hot air impingement blanching (HHAIB) for controlled polyphenol oxidase inactivation, antinutrient reduction, and structural quality preservation in yam. Food Control.

[b3] Anthon G.E., Barrett D.M. (2001). Colorimetric method for the determination of lipoxygenase activity. J. Agricult. Food Chem..

[b4] Anthon G.E., Barrett D.M. (2002). Kinetic parameters for the thermal inactivation of quality-related enzymes in carrots and potatoes. J. Agricult. Food Chem..

[b5] Astráin-Redín L., Raso J., Álvarez I., Kirkhus B., Meisland A., Borge G.I.A., Cebrián G. (2023). New pulsed electric fields approach to improve the blanching of carrots. Lwt.

[b6] Bahçeci K.S., Serpen A., Gökmen V., Acar J. (2005). Study of lipoxygenase and peroxidase as indicator enzymes in green beans: change of enzyme activity, ascorbic acid and chlorophylls during frozen storage. J. Food Eng..

[b7] Barrett D.M., Theerakulkait C. (1995). Quality indicators in blanched, frozen, stored vegetables. Food Technol. (Chicago).

[b8] Canet W., Alvarez M.D., Luna P., Fernández C., Tortosa M.E. (2005). Blanching effects on chemistry, quality and structure of green beans (cv. Moncayo). Eur. Food Res. Technol..

[b9] De Corcuera J.R., Cavalieri R., Powers J. (2003). Prototype instruments for laboratory and on-line measurement of lipoxygenase activity. Food Sci. Technol. Int..

[b10] Dermesonluoglu E., Katsaros G., Tsevdou M., Giannakourou M., Taoukis P. (2015). Kinetic study of quality indices and shelf life modelling of frozen spinach under dynamic conditions of the cold chain. J. Food Eng..

[b11] Fraeye I., De Roeck A., Duvetter T., Verlent I., Hendrickx M., Van Loey A. (2007). Influence of pectin properties and processing conditions on thermal pectin degradation. Food Chem..

[b12] Garrote R.L., Silva E.R., Bertone R.A. (2001). Kinetic parameters for thermal inactivation of cut green beans lipoxygenase calculated using unsteady-state methods. Int. J. Food Sci. Technol..

[b13] Gökmen V. (2010). Selection of the indicator enzyme for blanching of vegetables. Enzym. Fruit Veg. Process..

[b14] Gonçalves E.M., Pinheiro J., Alegria C., Abreu M., Brandao T.R., Silva C.L. (2009). Degradation kinetics of peroxidase enzyme, phenolic content, and physical and sensorial characteristics in broccoli (brassica oleracea l. ssp. italica) during blanching. J. Agricult. Food Chem..

[b15] Güneş B., Bayindirli A. (1993). Peroxidase and lipoxygenase inactivation during blanching of green beans, green peas and carrots. LWT-Food Sci. Technol..

[b16] Halpin B., Lee C. (1987). Effect of blanching on enzyme activity and quality changes in green peas. J. Food Sci..

[b17] Hendrickx M., Maesmans G., De Cordt S., Noronha J., Van Loey A., Tobback P., Paulson A.T. (1995). Evaluation of the integrated time-temperature effect in thermal processing of foods. Crit. Rev. Food Sci. Nutr..

[b18] Indrawati I., Ludikhuyze L.R., Van Loey A.M., Hendrickx M.E. (2000). Lipoxygenase inactivation in green beans (phaseolus v ulgaris l.) due to high pressure treatment at subzero and elevated temperatures. J. Agricult. Food Chem..

[b19] Iribe-Salazar R., Caro-Corrales J., Hernández-Calderón Ó., Zazueta-Niebla J., Gutiérrez-Dorado R., Carrazco-Escalante M., Vázquez-López Y. (2015). Heat transfer during blanching and hydrocooling of broccoli florets. J. Food Sci..

[b20] Jha P.K., Xanthakis E., Chevallier S., Jury V., Le-Bail A. (2019). Assessment of freeze damage in fruits and vegetables. Food Res. Int..

[b21] Jiang H., Ling B., Zhou X., Wang S. (2020). Effects of combined radio frequency with hot water blanching on enzyme inactivation, color and texture of sweet potato. Innov. Food Sci. Emerg. Technol..

[b22] Kunzek H., Müller S., Vetter S., Godeck R. (2002). The significance of physico chemical properties of plant cell wall materials for the development of innovative food products. Eur. Food Res. Technol..

[b23] Lamberg I., Hallström B. (1986). Thermal properties of potatoes and a computer simulation model of a blanching process. Int. J. Food Sci. Technol..

[b24] Mcdonald A., Bowman C. (2018).

[b25] Morales-Blancas E., Chandia V., Cisneros-Zevallos L. (2002). Thermal inactivation kinetics of peroxidase and lipoxygenase from broccoli, green asparagus and carrots. J. Food Sci..

[b26] Okonkwo C.E., Moses O.I., Nwonuma C., Abiola T., Benjamin B.O., Folorunsho J.O., Olaniran A.F., Pan Z. (2022). Infrared and microwave as a dry blanching tool for irish potato: Product quality, cell integrity, and artificial neural networks (ANNs) modeling of enzyme inactivation kinetic. Innov. Food Sci. Emerg. Technol..

[b27] Pérez-Calderón J., Califano A., Santos M.V., Zaritzky N. (2017). Kinetic parameters for the thermal inactivation of peroxidase and lipoxygenase in precooked frozen brassica species. J. Food Sci..

[b28] Pérez-Calderón J., Santos M.V., Zaritzky N. (2019). Processing of pre-cooked frozen Brussels sprouts: Heat transfer modelling as related to enzyme inactivation and quality stability. Food Bioprod. Process..

[b29] Pero M., Kiani H., Skåra T., Skipnes D., Askari G. (2019). Optimizing thermal processing of broccoli: model development, numerical simulation, experimental validation. Int. J. Food Eng..

[b30] Połata H., Wilińska A., Bryjak J., Polakovič M. (2009). Thermal inactivation kinetics of vegetable peroxidases. J. Food Eng..

[b31] Rayan A.M., Gab-Alla A.A., Shatta A.A., El-Shamei Z.A. (2011). Thermal inactivation kinetics of quality-related enzymes in cauliflower (brassica oleracea var. botrytis). Eur. Food Res. Technol..

[b32] Reyes-De-Corcuera J.I., Cavalieri R.P., Powers J.R. (2002). Improved amperometric method for the rapid and quantitative measurement of lipoxygenase activity in vegetable tissue crude homogenates. J. Agricult. Food Chem..

[b33] Richter Reis F. (2016). New Perspectives on Food Blanching.

[b34] Ruiz-Ojeda L.M., Peñas F.J. (2013). Comparison study of conventional hot-water and microwave blanching on quality of green beans. Innov. Food Sci. Emerg. Technol..

[b35] Van der Sman R. (2008). Prediction of enthalpy and thermal conductivity of frozen meat and fish products from composition data. J. Food Eng..

[b36] Van der Sman R. (2020). Impact of processing factors on quality of frozen vegetables and fruits. Food Eng. Rev..

[b37] van der Sman R., Guo X., Pedreschi R. (2026). Physics-informed neural network for prediction of post-harvest firmness of avocados. Postharvest Biology Technol..

[b38] van der Sman R., Schenk E. (2024). Causal factors concerning the texture of french fries manufactured at industrial scale. Curr. Res. Food Sci..

[b39] Soysal Ç., Söylemez Z. (2005). Kinetics and inactivation of carrot peroxidase by heat treatment. J. Food Eng..

[b40] Stolle-Smits T., Beekhuizen J.G., Recourt K., Voragen A.G., van Dijk C. (2000). Preheating effects on the textural strength of canned green beans. 1. cell wall chemistry. J. Agricult. Food Chem..

[b41] Van Buggenhout S., Sila D., Duvetter T., Van Loey A., Hendrickx M. (2009). Pectins in processed fruits and vegetables: Part III—Texture engineering. Compr. Rev. Food Sci. Food Saf..

[b42] Verlinden B.E., De Baerdemaeker J. (1997). Modeling low temperature blanched carrot firmness based on heat induced processes and enzyme activity. J. Food Sci..

[b43] Waldron K., Parker M., Smith A. (2003). Plant cell walls and food quality. Compr. Rev. Food Sci. Food Saf..

[b44] Weng Z., Hendrickx M., Maesmans G., Gebruers K., Tobback P. (1991). Thermostability of soluble and immobilized horseradish peroxidase. J. Food Sci..

[b45] Weng Z., Hendrickx M., Maesmans G., Tobback P. (1992). The use of a time-temperature-integrator in conjunction with mathematical modelling for determining liquid/particle heat transfer coefficients. J. Food Eng..

[b46] Williams D., Lim M., Chen A., Pangborn R., Whitaker J. (1986). Blanching of vegetables for freezing: which indicator enzyme to choose. Food Technol. (USA).

[b47] Wolf S., Greiner S. (2012). Growth control by cell wall pectins. Protoplasma.

[b48] Zhan X., Zhu Z., Sun D.-W. (2019). Effects of pretreatments on quality attributes of long-term deep frozen storage of vegetables: a review. Crit. Rev. Food Sci. Nutr..

